# Supplementary material for: Promoting Beneficial Arthropods in Urban Agroecosystems: Focus on Flowers, Maybe Not Native Plants
Source: Insects. 2023 Jun 23;14(7):576. doi: 10.3390/insects14070576 (PMC10380228; doi:10.3390/insects14070576)

insects

Article

Promoting beneficial arthropods in urban agroecosystems: focus on flowers, Maybe not native plants

Stacy M Philpott [1]*, Azucena Lucatero [1], Sofie Andrade [2], Cameron Hernandez [1], Peter Bichier[1]

[1] Environmental Studies Department, University of California, Santa Cruz, USA; sphilpot@ucsc.edu
[2] Ecology and Evolutionary Biology Department, University of California, Santa Cruz, USA
* Correspondence: sphilpot@ucsc.edu; Tel.: +01-831-459-1549

Supplementary Material

Table S1. List of ground-foraging spider, ladybeetle, bee and ant species encountered, abundance across all sites and years, and native status.

Species	No. indiv.	Native Status	Source
**ARANAE - SPIDERS**			
Amaurobiidae sp. 1	2	Likely Native	[66]
Amaurobiidae sp. 2	1	Likely Native	[66]
Anyphaenidae sp. 1	1	Unknown	[66]
Araneidae sp. 1	2	Unknown	[66]
Araneidae sp. 2	3	Unknown	[66]
Caponiidae sp. 1	7	Native	[66]
Clubionidae sp. 1	2	Unknown	[66]
Clubionidae sp. 2	1	Unknown	[66]
Corinnidae sp. 1	3	Unknown	[66]
Corinnidae sp. 2	1	Unknown	[66]
Corinnidae sp. 3	6	Unknown	[66]
Corinnidae sp. 4	1	Unknown	[66]
Corinnidae sp. 5	21	Unknown	[66]
Cybaeidae sp. 1	4	Likely Native	[66]
Dictynidae sp. 1	2	Likely Native	[66]
Drassyllus saphes	66	Native	[67]
Dysdera crocata	66	Not-Native	[66, 67]
Hahniidae sp. 1	2	Likely Native	[66]
Linyphiidae sp. 1	18	Likely Native	[66]
Linyphiidae sp. 2	3	Likely Native	[66]
Linyphiidae sp. 3	4	Likely Native	[66]
Micaria utahna	35	Native	[67]
Miturgidae sp. 1	3	Not-Native	[66]
Miturgidae sp. 2	1	Not-Native	[66]

Nesticidae sp. 1	41	Likely Native	[66]
Nesticidae sp. 2	60	Likely Native	[66]
Nesticidae sp. 3	1	Likely Native	[66]
Oecobiidae sp. 1	11	Unknown	[66]
Oecobiidae sp. 2	7	Unknown	[66]
Oonopidae sp. 1	1	Unknown	[66]
Oxyopidae sp. 1	1	Native	[66]
Pardosa sierra	976	Native	[67, 68]
Pholcidae sp. 1	2	Unknown	[66]
Pholcidae sp. 2	2	Unknown	[66]
Pimoidae sp. 1	2	Likely Native	[66]
Pimoidae sp. 2	1	Likely Native	[66]
Salticidae sp. 1	1	Unknown	[66]
Salticidae sp. 2	5	Unknown	[66]
Salticidae sp. 3	2	Unknown	[66]
Schizocosa mccooki	8	Native	[67, 68]
Theridiidae sp. 1	5	Unknown	[66]
Theridiidae sp. 2	1	Unknown	[66]
Thomisidae sp. 1	1	Likely Native	[66]
Thomisidae sp. 2	1	Likely Native	[66]
Trachyzelotes barbatus	81	Not-Native	[67, 68]
Urozelotes rusticus	67	Not-Native	[66, 67]
**COLEOPTERA: COCCINELLIDAE - LADYBEETLES**			
Adalia bipunctata	1	Native	[64]
Coccinella californica	26	Native	[64]
Coccinella septempunctata	17	Not-Native	[64]
Cycloneda munda	14	Native	[52]
Cycloneda polita	9	Native	[64]
Cycloneda sanguinea	35	Native	[64]
Harmonia axyridis	91	Not-Native	[64]
Hippodamia convergens	91	Native	[64]
Hyperaspis quadrioculata	12	Native	[64]
Hyperaspis sp. A	2	Native	[65]
Nephus binaevatus	12	Not-Native	[64]
Olla v-nigrum	1	Native	[64]
Psyllobora vigintimaculata	1109	Native	[64]
Scymnus cervicalis	1	Native	[64]
Scymnus coniferarum	2	Native	[64]
Scymnus margincollis	22	Native	[64]
Scymnus nebulosus	1	Native	[64]
Scymnus sp. 1	3	Native	[65]
Scymnus sp. A	5	Native	[65]

Stethorus punctum	112	Native	[64]
**HYMENOPTERA - BEES**			
Agapostemon sp. A	3	Likely Native	[61, 62]
Agapostemon texanus	150	Native	[61]
Andrena sp. 1	1	Likely Native	[62]
Andrena sp. 2	3	Likely Native	[62]
Andrena sp. A	1	Likely Native	[62]
Anthidium manicatum	2	Native	[62]
Anthophora urbana	7	Native	[62]
Anthophora sp. 1	2	Likely Native	[62]
Anthophora sp. 2	1	Likely Native	[62]
Anthophora sp. A	1	Likely Native	[62]
Apis mellifera	2544	Not-Native	[62]
Ashmeadiella bucconis	6	Native	[61]
Ashmeadiella sp. 1	1	Native	[61]
Bombus californicus	1	Native	[62]
Bombus caliginosus	153	Native	[62]
Bombus vosnesenskii	224	Native	[62]
Ceratina acantha	70	Native	[61]
Ceratina nanula	27	Native	[62]
Ceratina sequoiae	1	Native	[62]
Ceratina sp. 1	21	Likely Native	[61, 62]
Ceratina sp. 2	10	Likely Native	[61, 62]
Coelioxys rufitarsis	2	Native	[62]
Coelioxys sp. 1	1	Likely Native	[62]
Colletes sp. A	7	Likely Native	[62]
Diadasia sp. 1	3	Native	[61]
Dufourea sp. A	1	Likely Native	[61]
Eucera actuosa	4	Native	[62]
Eucera sp. A	1	Likely Native	[62]
Halictus confusus	3	Native	[61]
Halictus farinosus	11	Native	[62]
Halictus ligatus	79	Native	[61]
Halictus rubicundus	4	Native	[62]
Halictus tripartitus	1328	Native	[61]
Halictus sp. 1	1	Native	[61]
Halictus sp. 2	4	Native	[61]
Halictus sp. A	4	Native	[61]
Holcopasites sp. A	1	Native	[62]
Hylaeus leptocephalus	21	Not-Native	[62]
Hylaeus mesillae	106	Native	[62]
Hylaeus panamensis	13	Native	[62]

Hylaeus polifolii	7	Native	[63]
Hylaeus punctatus	58	Not-Native	[63]
Hylaeus rudbeckiae	98	Native	[62]
Hylaeus sp. 1	34	Unknown	NA
Hylaeus sp. 2	3	Unknown	NA
Hylaeus sp. 3	2	Unknown	NA
Hylaeus sp. A	6	Unknown	NA
Hylaeus sp. B	1	Unknown	NA
Hylaeus sp. C	12	Unknown	NA
Lasioglossum (Dialictus) incompletum	1	Likely Native	[63]
Lasioglossum (Dialictus) sp. A	139	Likely Native	[62]
Lasioglossum (Evylaeus) sp. A	7	Likely Native	[62]
Lasioglossum (Lasioglossum) sp. A	39	Likely Native	[62]
Lasioglossum sp. 1	4	Likely Native	[62]
Lasioglossum sp. 2	3	Likely Native	[62]
Lasioglossum sp. 3	13	Likely Native	[62]
Lasioglossum sp. 4	1	Likely Native	[62]
Lasioglossum sp. 5	10	Likely Native	[62]
Lasioglossum sp. 6	7	Likely Native	[62]
Lasioglossum sp. 7	2	Likely Native	[62]
Lasioglossum sp. 8	2	Likely Native	[62]
Lasioglossum sp. 9	2	Likely Native	[62]
Lasioglossum sp. 10	1	Likely Native	[62]
Lasioglossum sp. 11	1	Likely Native	[62]
Lasioglossum sp. 12	4	Likely Native	[62]
Lasioglossum sp. 13	5	Likely Native	[62]
Lasioglossum sp. 14	111	Likely Native	[62]
Lasioglossum sp. 15	4	Likely Native	[62]
Lasioglossum sp. A	43	Likely Native	[62]
Martinapis sp. 1	1	Likely Native	[62]
Megachile apicalis	1	Not-Native	[63]
Megachile brevis	4	Native	[62]
Megachile latimanus	1	Native	[62]
Megachile mendica	1	Native	[62]
Megachile perihirta	30	Native	[61]
Megachile relativa	1	Native	[62]
Megachile rotundata	7	Not-Native	[61]
Megachile sp. 1	1	Unknown	NA
Megachile sp. 2	3	Unknown	NA
Megachile sp. 3	7	Unknown	NA
Megachile sp. 4	1	Unknown	NA
Megachile sp. 5	1	Unknown	NA

Megachile sp. A	1	Unknown	NA
Megachile sp. B	2	Unknown	NA
Melissodes robustior	100	Native	[61]
Melissodes sp. 1	27	Native	[61]
Melissodes sp. 2	2	Native	[61]
Melissodes sp. A	1	Native	[61]
Melissodes sp. B	8	Native	[61]
Nomada edwardsii	2	Native	[61]
Nomada sp. A	4	Likely Native	[62]
Osmia sp. 1	1	Likely Native	[62]
Peponapis pruinosa	7	Native	[61]
Peponapis sp. A	1	Native	[61, 62]
Sphecodes sp. 1	3	Unknown	NA
Sphecodes sp. A	3	Unknown	NA
Svastra aegis	2	Native	[61]
Xylocopa tabaniformis	36	Native	[61]
Xylocopa varipuncta	47	Native	[62]
Unknown Unknown	4	Unknown	NA
**HYMENOPTERA: FORMICIDAE - ANTS**			
Cardiocondyla mauritanica	123	Not-Native	[69]
Formica moki	1	Native	[69]
Hypoponera opacior	106	Native	[69]
Linepithema humile	369	Not-Native	[59]
Nylanderia vividula	36	Not-Native	[59]
Prenolepis imparis	5	Native	[69]
Tapinoma sessile	11	Native	[69]
Tetramorium caespitum	75	Not-Native	[59]

Table S2. List of all native plant species encountered in urban community gardens in the California central coast, and their relative abundance as a fraction of total and native plant species cover.

**Family**	**Genus**	**Species**	**Species or morphospecies or common name**	**% of total native cover**	**% of all plant cover**
Amaranthaceae	*Amaranthus*	*palmeri*	Pigweed	3.57	0.09
Apocynaceae	*Asclepias*	*fascicularis*	Milkweed	0.22	0.01
Asteraceae	*Achillea*	*millefolium*	Yarrow	5.58	0.14
Asteraceae	*Cirsium*	*douglasii*	Thistle	1.34	0.03
Asteraceae	*Erigeron*	*canadensis*	Horseweed	1.23	0.03
Asteraceae	*Helianthus*	*annuus*	Sunflower	39.04	0.99
Asteraceae	*Matricaria*	*discoidea*	Pineapple weed	0.17	0.00
Boraginaceae	*Phacelia*	*sp.*	Scorpionweed	1.67	0.04
Boraginaceae	*Plagiobothrys*	*sp.*	Popcorn flower	2.51	0.06
Brassicaceae	*Cardamine*	*oligosperma*	Popweed	0.17	0.00
Brassicaceae	*Nasturtium*	*officinale*	Watercress	1.51	0.04
Equisetaceae	*Equisetum*	*sp.*	Horsetail	0.84	0.02
Lamiaceae	*Salvia*	*mellifera*	Black sage	1.56	0.04
Montiaceae	*Claytonia*	*sp.*	Miner's lettuce	0.11	0.00
Onagraceae	*Clarkia*	*sp.*	Clarkia	0.73	0.02
Onagraceae	*Epilobium*	*sp.*	Fireweed	3.90	0.10
Orobanchaceae	*Castilleja*	*exserta*	Owl's clover	0.06	0.00
Papaveraceae	*Eschscholzia*	*californica*	California poppy	28.56	0.72
Pteriadceae	*Adiantum*	*sp.*	Maiden-hair fern	0.11	0.00
Ranunculaceae	*Delphinium*	*sp.*	Larkspur	7.14	0.18

Table S3. Model output from GLMM (bees and ladybeetles) and GLM (spiders and ants) models testing relationships between arthropod and native arthropod abundance and richness and local and landscape features of urban agroecosystems.

Dependent variable	Model reported	Predictor Variables in Model	No. of models	Estimate	z or t score*	p-value
LNBeeIndiv	best	(Intercept)	1	4.956	14.728	<0.001
		LNGardenSize	1	0.297	3.719	<0.001
LNNumNativeLikelyNativeBeeIndiv	average	(Intercept)	4	4.301	11.937	<0.001
		LNGardenSize	2	0.303	2.349	0.019
		LNNumNativePlantSpp	2	-0.392	1.601	0.1094
LNNumNativeBeeIndiv	best	(Intercept)	4	4.154	9.023	<0.001
		LNGardenSize	2	0.333	2.351	0.018
		LNNumNativePlantSpp	2	-0.475	1.777	0.076
NumNonNativeBeeIndiv	average	(Intercept)	2	3.737	7.421	<0.001
		Natural2km	2	0.016	4.176	<0.001
		LNGardenSize	1	0.203	2.235	0.0254
NumBeeSpp	average	(Intercept)	3	15.21	6.296	<0.001
		LNGardenSize	3	2.056	2.987	0.003
		LNNumNativePlantSpp	1	-0.81	0.627	0.53
		LNNumTreesShrubs	1	0.074	0.122	0.903
NumNativeLikelyNativeBeeSpp	average	(Intercept)	2	12.008	6.05	<0.001
		LNGardenSize	2	1.806	3.338	<0.001
		LNNumNativePlantSpp	1	-0.554	0.542	0.587
NumNativeBeeSpp	average	(Intercept)	2	8.135	3.946	<0.001
		LNGardenSize	2	1.525	3.007	0.003
		LNNumNativePlantSpp	1	-0.009	0.01	0.992
NumNonNativeBeeSpp	best	(Intercept)	1	0.461	2.236	0.175
LNNumLBIndiv	average	(Intercept)	2	3.566	13.268	<0.001
		Natural2km	1	-0.018	3.124	0.002
LNNumNativeLBIndiv	best	(Intercept)	1	3.3695	17.15	<0.001
LNNonNativeLBIndiv	best	(Intercept)	1	1.132	8.333	0.013
NumLBSpp	average	(Intercept)	3	4.614	4.28	<0.001
		LNNumTreesShrubs	3	1.038	3.393	<0.001
		Natural2km	3	-0.061	3.987	<0.001
		LNNumNativePlantSpp	1	-0.482	0.787	0.431
		LNNumFlowers	1	-0.391	1.195	0.231
NumNativeLBSpp	average	(Intercept)	3	3.963	5.736	<0.001
		LNNumTreesShrubs	2	0.558	1.885	0.059
		Natural2km	1	-0.042	3.28	0.001
		LNNumNativePlantSpp	1	-0.041	0.074	0.941
NonNativeLBSpp	best	(Intercept)	1	1.0811	8.271	0.032

LNNumSpiders	average	(Intercept)	6	3.655	4.816	<0.001
		LNGardenSize	2	0.477	1.684	0.092
		LNNumFlowers	2	0.216	1.244	0.214
		Mulch1m	1	-0.018	1.263	0.207
		Natural2km	1	0.011	0.917	0.359
LNNumNativeLikelyNativeSpiders	average	(Intercept)	5	3.529	6.063	<0.001
		Mulch1m	2	-0.029	1.529	0.126
		LNGardenSize	1	0.446	1.195	0.232
		Natural2km	1	0.015	1.017	0.309
LNNumNativeSpiders	average	(Intercept)	3	2.961	5.06	<0.001
		Natural2km	1	0.021	1.195	0.232
		Mulch1m	1	-0.02	0.869	0.385
NonNativeSpiderIndiv	best	(Intercept)	1	-6.057	-0.795	0.438
		LnNumFlowers	1	3.488	2.395	0.028
NumSpiderSpp	average	(Intercept)	2	1.739	6.135	<0.001
		LNNumFlowers	2	0.123	2.344	0.019
		LNGardenSize	1	0.127	1.299	0.194
NumNativeLikelyNativeSpiderSpp	average	(Intercept)	2	1.473	3.933	<0.001
		LNNumFlowers	1	0.099	1.434	0.151
NumNativeSpiderSpp	average	(Intercept)	5	1.804	2.068	0.039
		Natural2km	2	0.022	1.531	0.126
		LNNativePlantSpp	2	-1.459	2.036	0.042
		LNNumFlowers	2	0.474	1.988	0.047
		LNNumTreesShrubs	1	0.405	1.407	0.159
NonNativeSpiderSpp	average	(Intercept)	2	0.321	0.429	0.66
		LNGardenSize	2	0.903	4.1	<0.001
		LNNumTreesShrubs	1	0.3	2.038	0.041
		Mulch1m	2	0.023	2.053	0.04
		LNNumFlowers	2	0.279	2.403	0.016
NumAntOccur	average	(Intercept)	3	3.562	44.037	<0.001
		Mulch1m	1	0.004	1.888	0.059
		LNNumTreesShrubs	1	0.054	1.484	0.138
NumNativeAntOccur	best	(Intercept)	1	1.477	7.341	<0.001
		Natural2km	1	0.017	2.478	0.024
LNNumNonNativeAntOccur	average	(Intercept)	5	3.509	14.488	<0.001
		LNNumFlowers	2	-0.071	1.419	0.156
		Mulch1m	2	0.005	1.296	0.195
		Natural2km	1	-0.003	0.922	0.356
NumAntSpp	best	(Intercept)	1	3.579	12.82	<0.001
NumNativeAntSpp	best	(Intercept)	1	1.421	8.945	<0.001

NumNonNativeAntSpp	average	(Intercept)	3	2.401	4.234	<0.001
		LNNumFlowers	1	-0.16	1.067	0.286
		Natural2km	1	-0.009	0.847	0.397
Num*Linepithema.humile*	average	(Intercept)	3	17.507	3.773	<0.001
		LNNumTreesShrubs	1	3.074	1.408	0.159
		LNGardenSize	1	-2.503	0.897	0.370
Num*Harmonia.axyridis*	average	(Intercept)	2	1.247	5.922	<0.001
		Natural2km	2	-0.015	2.507	0.012
		Mulch1m	1	-0.007	1.014	0.310

* z-score for averaged models, t-score for best models

Figure S1. Photo of a sticky trap and a pitfall trap before placing the cover (A) and a pitfall trap with the green plastic plate cover (B) in urban garden study sites.

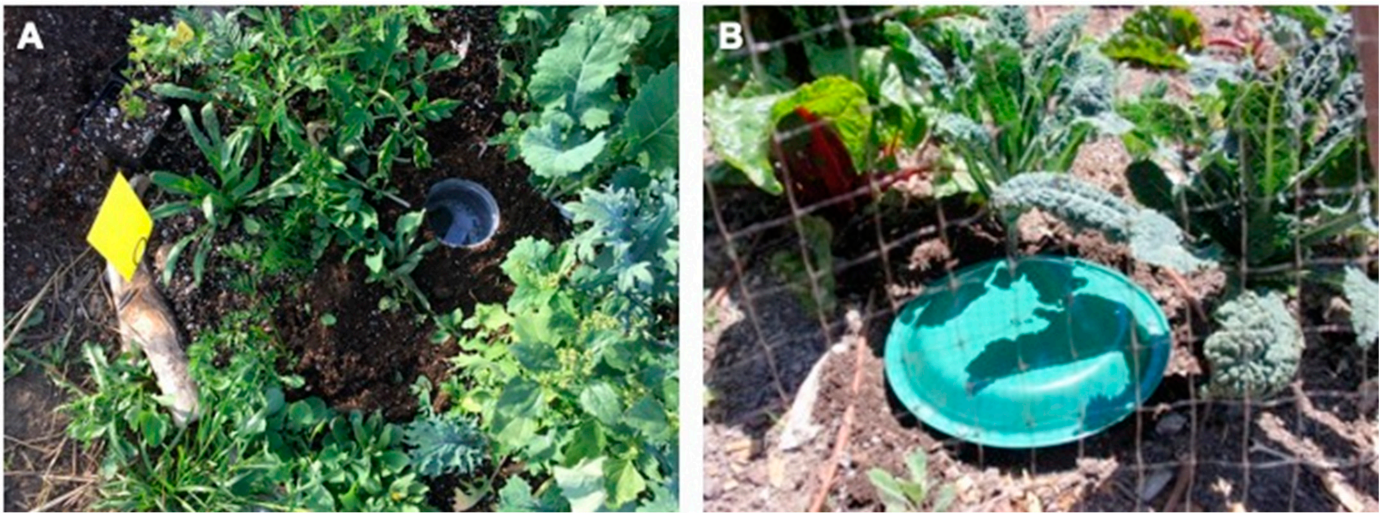

Figure S2. Significant relationships between abundance of the non-native ladybug species Harmonia axyridis and one landscape factor (natural habitat cover within 2 km) in urban gardens in the California Central Coast. The relationship is significant at P=0.012).

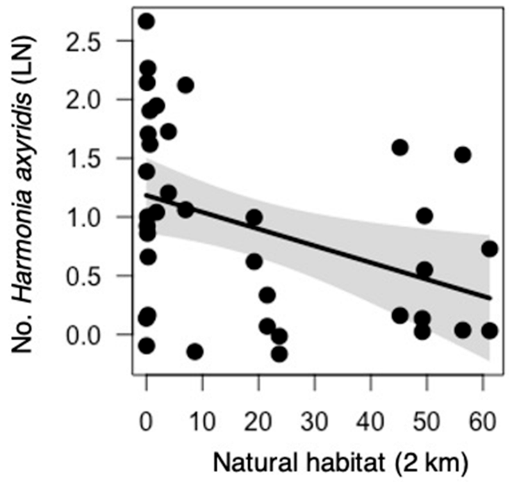

Supplement: Supplementary file 1 [file insects-14-00576-s001.zip › insects-2433632-supplementary.pdf]
